# Tracking of overweight and obesity from early childhood to adolescence in a population-based cohort – the Tromsø Study, *Fit Futures*

**DOI:** 10.1186/s12887-016-0599-5

**Published:** 2016-05-10

**Authors:** Elin Evensen, Tom Wilsgaard, Anne-Sofie Furberg, Guri Skeie

**Affiliations:** Clinical Research Department, University Hospital of North Norway, Tromsø, Norway; Department of Community Medicine, Faculty of Health Sciences, UiT The Arctic University of Norway, Tromsø, Norway; Department of Microbiology and Infection Control, University Hospital of North Norway, Tromsø, Norway

**Keywords:** Overweight, Obesity, Prevalence, Tracking, Childhood, Adolescence, Norway

## Abstract

**Background:**

Obesity is a serious childhood health problem today. Studies have shown that overweight and obesity tend to be stable (track) from birth, through childhood and adolescence, to adulthood. However, existing studies are heterogeneous; there is still no consensus on the strength of the association between high birth weight or high body mass index (BMI) early in life and overweight and obesity later in life, nor on the appropriate age or target group for intervention and prevention efforts. This study aimed to determine the presence and degree of tracking of overweight and obesity and development in BMI and BMI standard deviation scores (SDS) from childhood to adolescence in the *Fit Futures* cohort from North Norway.

**Methods:**

Using a retrospective cohort design, data on 532 adolescents from the *Fit Futures* cohort were supplemented with height and weight data from childhood health records, and BMI was calculated at 2–4, 5–7, and 15–17 years of age. Participants were categorized into weight classes by BMI according to the International Obesity Taskforce’s age- and sex-specific cut-off values for children 2–18 years of age (thinness: adult BMI <18.5 kg/m^2^, normal weight: adult BMI ≥18.5- < 25 kg/m^2^, overweight: adult BMI ≥25- < 30 kg/m^2^, obesity: adult BMI ≥30 kg/m^2^). Non-parametric tests, Cohen’s weighted Kappa statistic and logistic regression were used in the analyses.

**Results:**

The prevalence of overweight and obesity combined, increased from 11.5 % at 2–4 years of age and 13.7 % at 5–7 years of age, to 20.1 % at 15–17 years of age. Children who were overweight/obese at 5–7 years of age had increased odds of being overweight/obese at 15–17 years of age, compared to thin/normal weight children (crude odds ratio: 11.1, 95 % confidence interval: 6.4–19.2). Six out of 10 children who were overweight/obese at 5–7 years of age were overweight/obese at 15–17 years of age.

**Conclusions:**

The prevalence of overweight and obesity increased with age. We found a moderate indication of tracking of overweight/obesity from childhood to adolescence. Preventive and treatment initiatives among children at high risk of overweight and obesity should start before 5–7 years of age, but general preventive efforts targeting all children are most important.

**Electronic supplementary material:**

The online version of this article (doi:10.1186/s12887-016-0599-5) contains supplementary material, which is available to authorized users.

## Background

Globally obesity has more than doublet since 1980 [1]. The World Health Organization, Europe has reported that about 20 % of children and adolescents were overweight, and a third of these were obese (2007 figures) [2]. Recent research suggests that the prevalence of overweight and obesity in children is plateauing [3]. In Norway, the prevalence of overweight and obesity combined was 15.8 % in 8–9-year-old children in 2012, when a plateau might have been reached [4]. Overall Norway and the Nordic countries experience lower rates of overweight and obesity among children and adolescents than other countries in Europe and the United States [2]. Other studies have reported a higher prevalence of overweight and obesity in children from the northernmost region of Norway [4–6]. Plateau or not, the prevalence of overweight and obesity is still high, and obesity is regarded as one of the most serious childhood health problem globally [1, 7]. Obesity in childhood or adolescence is associated with a higher risk of weight-related morbidity and premature mortality in adulthood [8, 9]. A recently published review and meta-analysis [10] state that childhood body mass index (BMI) is not a good predictor of adult disease, since most obesity-related adult morbidity occurs in adults with a childhood BMI within the normal range.

Several studies have shown that overweight and obesity tend to be stable over an individual’s lifetime, from childhood to adolescence, and in some studies also into adulthood [10–14]. The stability of a risk factor over time, or the maintenance of a relative position within a distribution of values over time, is known as tracking, which can also be defined as the predictability of future values of a risk factor based on earlier measurements [15, 16]. Many recent studies have focused on birth weight and growth patterns in early infancy as determinants of overweight and obesity later in life [17–23]. However, these studies are heterogeneous: follow-up time varies and different definitions of overweight and obesity and rapid growth were used, which makes it difficult to draw firm conclusions [7, 24]. Thus, there is still no consensus on the strength of the association between high birth weight or high BMI early in life and overweight and obesity later in life.

Although several researchers have expressed the need for early preventive efforts and intervention to stem the tide of overweight and obesity [7, 23, 24], exactly how early intervention should take place and if overweight or obese children should be the main target for interventions, is still a matter of discussion. Moreover, assessing the efficacy of interventions and preventive efforts can be challenging, as discussion regarding the best measure of adiposity change in children is on-going [25, 26], and there is limited knowledge on the natural development of BMI in overweight and obese children.

The aim of this study was to investigate how important early childhood overweight and obesity is in relation to overweight and obesity at adolescence. Specifically, we wanted to 1) examine the prevalence of overweight and obesity at different ages, 2) determine the presence and degree of tracking of overweight and obesity from different ages in childhood to adolescence, and 3) investigate any differences in the natural development of BMI and BMI standard deviation scores (SDS) in overweight and obese children vs. thin and normal weight children in a cohort of youths from an area with an above average prevalence of overweight and obesity.

## Methods

### Study sample

**Fig. 1:**
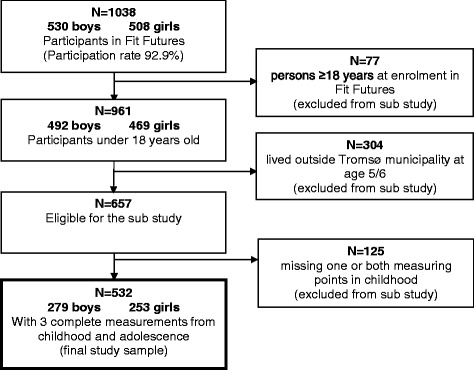
Flowchart of the study sample. The Tromsø Study: *Fit Futures 1* 2010/2011

*Fit Futures* is a cohort of adolescents and part of The Tromsø Study, a population-based health study focusing on somatic health and lifestyle measurements. *Fit Futures 1* was conducted in the 2010/2011 school year. All students from the Tromsø region, North Norway, in their first year of upper-secondary school was invited (*n* = 1117, mainly aged 15–19 years). A total of 1038 students participated in *Fit Futures 1* (participation proportion 92.9 %). Detailed information on the *Fit Futures 1* study and the cohort has been presented in an earlier paper [27]. For the present study, all students over 18 years of age were excluded (*n* = 77), and for practical reasons students who did not grow up in the Tromsø municipality were also excluded (*n* = 304), leaving 657 eligible youths. The data from *Fit Futures 1* were then supplemented with height and weight data from two time points during childhood for these youths; complete sets of measurements were available for 532 of them (279 boys and 253 girls), who constituted the final study sample (Fig. 1).

The Norwegian Data Inspectorate and The Regional Committee for Medical and Health Research Ethics, North Norway (REC North) approved *Fit Futures 1*. Written informed consent was obtained from all students. For students under 16 years of age, additional written consent from parents/guardians was obtained. The present study was also approved by REC North (Reference number: 2011/1284/REK nord).

### Data/measurements

In *Fit Futures 1*, anthropometric measures were taken by specially trained nurses, following standardized procedures. Participants were measured wearing light clothing and no footwear. Height and weight were measured to the nearest 0.1 cm and 0.1 kg, respectively, on an automatic electronic scale (Jenix DS 102 stadiometer, Dong Sahn Jenix, Seoul, Korea). Additional data on height, weight measurements, age, and date of measurements were collected from childhood health records using the unique personal identification number of each youth. In Norway, regular health controls by public health nurses including measurement of height and weight are offered for all children (voluntary) from birth through school age in accordance with national preventive health programme guidelines. For these children, born 1992–1994, health controls were offered at 2 and 6 years of age. If data were missing for the exact age or a child had several measurements in the period around 2–4 years or 5–7 years, the measurement closest to the 2-year or 6-year birthday, respectively, was recorded.

BMI was calculated as weight in kg divided by height in meters squared, (kg/m^2^) and was used to categorize participants into weight classes according to the International Obesity Taskforce (IOTF) age- and sex-specific international cut-off values for children 2–18 years of age [28]. Age at last birthday and reference values for BMI at midyear were used to classify the participants at all three ages [29]. The following four weight classes were used: thinness (corresponds to an adult BMI <18.5 kg/m^2^), normal weight (corresponds to an adult BMI ≥18.5- < 25 kg/m^2^), overweight (corresponds to an adult BMI ≥25- < 30 kg/m^2^) and obesity (corresponds to an adult BMI ≥30 kg/m^2^). As there were few thin and obese participants, weight classes were merged into thinness/normal weight and overweight/obesity in some of the statistical analyses. BMI SDS was calculated using the LMS (median (M), variation (S) and skewness (L)) method, and the BMI LMS coefficients corresponding to the IOTF cut-off values [28] and the United Kingdom (UK) reference population from 1990 [30].

### Statistical analyses

Statistical analyses were carried out using IBM SPSS® for Windows, version 21/22. If not otherwise specified, the level of statistical significance was set to two-sided p-values <0.05.

Weight and BMI data in this sample were not normally distributed. Therefore data were analysed using non-parametric tests and logistic regression. Development in mean BMI and BMI SDS from childhood to adolescence was analysed using Friedman’s ANOVA for repeated measures. Post hoc tests were conducted using the Mann–Whitney *U* test for comparing different groups. A stricter confidence level of 99 % was used in these tests, to correct for repeated measures. Gender differences were examined by Chi square tests. Positive predictive values were calculated between pair-wise time points.

Tracking was determined by the odds ratio (OR) of being overweight/obese at 5–7 years of age, or at 15–17 years of age according to weight class at 2–4 years of age or 5–7 years of age. The merged weight classes thinness/normal weight and overweight/obesity were used as dependent and independent categorical variables in logistic regression and the non-parametric tests. Exact age at the time of childhood measurements, gender, and time between measurements (in years, months) were included as covariates. Interaction terms between gender and weight class at 2–4 and 5–7 years of age were assessed in separate models. None of these terms were significant and were therefore not included in our final models.

A four-level Cohen’s weighted Kappa statistic was also used in the tracking analyses. The proportion of agreement was calculated, i.e. the proportion of children that remained in their weight class between ages. The observed proportions of agreement were compared with the proportions expected given no tracking (assuming the same distribution at both time points). Weighted Kappa analyses are not directly available in SPSS®, but an extension command, Stats Weighted Kappa.spe, available at the IBM® Support Portal [31] was used with a set of weights that are based on the squared distance between categories [32, 33]. We used the guidelines by Muñoz and Bangdiwala to interpret the Kappa coefficients [34].

A logistic regression model was used to estimate the probability (with 95 % confidence intervals (CI)) of being overweight/obese at 15–17 years of age based on BMI values at 2–4 or 5–7 years of age. Age and gender were included in the models and the probabilities of being overweight/obese at 15–17 years of age were estimated using mean values of age and gender in the models. The chosen BMI values for boys and girls correspond to the cut-off points of childhood BMI at 2.5 and 6.0 years of age corresponding to adult BMI category limits 18.5–35 kg/m^2^ [28].

## Results

### Prevalence of overweight and obesity

Mean BMI decreased between 2–4 years of age and 5–7 years of age and increased at 15–17 years of age. No significant gender difference in mean BMI was found at any age (Table 1). The majority (>70 %) of both boys and girls were categorized as normal weight at all ages. The prevalence of overweight and obesity combined increased with age and was 11.5 % at 2–4 years of age, 13.7 % at 5–7 years of age, and 20.1 % at 15–17 years of age for boys and girls combined. The prevalence of obesity alone was 1.5 %, 4.3 %, and 6.2 % at 2–4, 5–7, and 15–17 years of age, respectively (Table 2). No significant gender difference was found in the prevalence of overweight and obesity combined, except at 5–7 years of age, when almost twice as many girls (18.2 %) as boys (9.7 %) were classified as either overweight or obese (*p* < 0.01).

**Table 1:** Descriptive characteristics of the study sample^a^ at three ages

Age (years)	Gender	Age (years) Mean (SD)	Height (cm) Mean (SD)	Weight (kg) Mean (SD)	BMI (kg/m^2^) Mean (SD)	BMI SDS^b^ Mean (SD)
2–4	Boys	2.6 (0.23)	93.0 (3.91)**	14.1 (1.63)**	16.33 (1.35)	0.01 (1.01)
	Girls	2.6 (0.20)	91.3 (3.50)**	13.5 (1.57)**	16.19 (1.38)	0.08 (1.00)
5–7	Boys	6.0 (0.36)	117.8 (5.09)**	22.0 (3.56)**	15.77 (1.77)	0.14 (0.94)^‡^
	Girls	6.0 (0.38)	116.1 (5.03)**	21.6 (4.02)**	15.96 (2.10)	0.32 (1.06)^‡^
15–17	Boys	16.0 (0.39)	177.0 (6.64)**	69.5 (14.75)**	22.13 (4.29)	0.49 (1.12)
	Girls	16.1 (0.37)	165.1 (6.35)**	60.6 (11.82)**	22.22 (4.14)	0.40 (1.03)

^a^A sub study of The Tromsø Study: *Fit Futures N* = 532: 279 boys, 253 girls

** Statistically significant gender differences (Mann–Whitney *U* test *p* <0.001)

^‡^Statistically significant gender difference (Mann–Whitney *U* test *p* = 0.03)

^b^BMI SDS is calculated using LMS values based on an international reference population of children [28]

*BMI* body mass index, *SDS* standard deviation score, *SD* ± standard deviation, *LMS* LMS curves/ LMS method: median (M), coefficient of variation (S) and skewness (L)

**Table 2:** Prevalence of weight classes^a^ in the study sample^b^ at three ages

Age (years)	Gender	Thinness	Normal weight	Overweight	Obesity	Gender difference^c^
		% (n)	% (n)	% (n)	% (n)	p-value
2–4	Boys	14.7 (41)	76.7 (214)	7.2 (20)	1.4 (4)	0.09
	Girls	14.6 (37)	70.8 (179)	13.0 (33)	1.6 (4)	
5–7	Boys	7.5 (21)	82.8 (231)	6.5 (18)	3.2 (9)	<0.01
	Girls	10.7 (27)	71.1 (180)	12.6 (32)	5.5 (14)	
15–17	Boys	9.7 (27)	69.9 (195)	13.3 (37)	7.2 (20)	0.25
	Girls	5.9 (15)	74.3 (188)	14.6 (37)	5.1 (13)	

^a^Weight classes is based on BMI according to the International Obesity Taskforce’s age- and sex-specific cut-off values in children 2–18 years: thinness: adult BMI <18.5 kg/m^2^, normal weight: adult BMI ≥18.5- < 25 kg/m^2^, overweight: adult BMI ≥25- < 30 kg/m^2^, obesity: adult BMI ≥30 kg/m^2^ [28]

^b^A sub study of The Tromsø Study: *Fit Futures N* = 532: 279 boys, 253 girls

^c^Pearson’s Chi-Square tests for gender differences in weight classes were performed in a 2x3 contingency table with weight class overweight/obesity merged due to few obese participants

*BMI* body mass index

### Tracking of overweight/obesity

Tracking of overweight/obesity was present and significant between both 2–4 and 5–7 years of age, and between both of these ages and 15–17 years of age (Table 3). Mean time interval between measurements varied from 3.4 years for the first interval to 10.6 years for the second interval.

**Table 3:** Percentage of agreement and associations (ORs) between weight classes^a^ at different ages

		Overweight/obesity at 5–7 years					Overweight/obesity at 15–17 years				
			Crude		Adjusted^b^			Crude		Adjusted^b^	
Age (years)	Weight class^a^	%^c^	OR	95 % CI	OR	95 % CI	%^c^	OR	95 % CI	OR	95 % CI
2–4	Thin/normal weight		1.0		1.0			1.0		1.0	
	Overweight/obese	52.5	11.6	(6.4–21.0)	11.0^‡^	(6.0–20.1)	39.3	3.0	(1.7–5.3)	3.2	(1.8–5.6)
5–7	Thin/normal weight		-	-	-	-		1.0		1.0	
	Overweight/obese		-	-	-	-	63.0	11.1	(6.4–19.2)	12.1	(6.9–21.4)

A sub study of The Tromsø Study: *Fit Futures N* = 532: 279 boys, 253 girls

^a^Weight classes are based on BMI according to the International Obesity Taskforce’s age- and sex-specific cut-off values in children 2–18 years: thinness: adult BMI <18.5 kg/m^2^, normal weight: adult BMI ≥18.5- < 25 kg/m^2^, overweight: adult BMI ≥25- < 30 kg/m^2^, obesity: adult BMI ≥30 kg/m^2^ [28]

^b^Adjusted for gender, exact age (years, months) at first time point and time (years, months) between measurements

^c^Percent of those overweight/obese at younger age that are still overweight at the older age = percent agreement/ positive predictive value

^‡^ Gender was a significant covariate *p* = 0.02

*BMI* body mass index, *CI* confidence interval, *OR* odds ratio

The odds that a child that was overweight/obese at 5–7 years of age would be overweight/obese at 15–17 years of age was 11 times higher (95 % CI: 6.4–19.2) than those of a thin/normal weight child. A child that was overweight/obese at 2–4 years of age had odds of being overweight/obese at 15–17 years of age that were 3 times higher (95 % CI: 1.7–5.3) than those of a thin/normal weight child. Adjustments for covariates had only minor effects on the estimates and only gender between 2–4 and 5–7 years of age were significant. Expressed in other terms, 63.0 % (positive predictive value) of overweight/obese children stayed overweight/obese between 5–7 years of age and 15–17 years of age. Only 39.3 % of children who were overweight/obese at 2–4 years of age stayed overweight/obese at 15–17 years of age (Table 3).

Weighted Kappa statistic gave a similar result. From 2–4 years of age to 5–7 years of age *K*_*w*_ = 0.48 (99 % CI: 0.37–0.60) as well as from 5–7 years of age to 15–17 years of age *K*_*w*_ = 0.49 (99 % CI: 0.37–0.60), which is considered a moderate to substantial agreement. From 2–4 years of age to 15–17 years of age *K*_*w*_ = 0.22 (99 % CI: 0.11–0.33), which is considered a fair to moderate agreement.

The proportion of overweight/obese children that became thin/normal weight from 2–4 and 5–7 years of age to 15–17 years of age was 60.7 % and 37.0 %, respectively. The proportion of thin/normal weight children that became overweight/obese over the same time intervals was 17.6 % and 13.3 %, respectively. Among those who were overweight/obese at age 15–17 years, 22.4 % and 43.0 % were overweight/obese already at age 2–4 and 5–7 years, respectively.

### Prediction of overweight/obesity

Overall the estimated probability of being overweight/obese at 15–17 years of age based on BMI at 2.5 and 6 years of age, increased with increasing BMI and age (Table 4). The estimated probability of being overweight/obese at 15–17 years of age for a 2.5 year old with a BMI of 19.73 kg/m^2^ (boys) or 19.57 kg/m^2^ (girls) corresponding to an adult BMI of 30 kg/m^2^ was 0.54 (95 % CI: 0.40–0.67) for boys and 0.52 (95 % CI: 0.39–0.65) for girls. The estimated probability of being overweight/obese at 15–17 years of age for a 6.0 year old with a BMI of 19.76 kg/m^2^ (boys) or 19.61 kg/m^2^ (girls) corresponding to an adult BMI of 30 kg/m^2^, was 0.78 (95 % CI: 0.66–0.87) for boys and 0.76 (95 % CI: 0.63–0.86) for girls. BMI at 6-years of age corresponding to adult BMI of 27 kg/m^2^ or higher, predicted overweight/obesity at adolescence with probabilities higher than 50 %.

**Table 4:** Probabilities of overweight/obesity at 15–17 years (adult BMI ≥ 25 kg/m^2^), based at BMI at 2–4 and 5–7 years

		Boys			Girls		
Age (Years)	Corresponding adult BMI	Child BMI	P	95 % CI	Child BMI	P	95 % CI
2.5	18.5	15.02	0.11	(0.08–0.15)	14.77	0.10	(0.07–0.14)
	23.0	17.28	0.27	(0.23–0.32)	17.01	0.24	(0.21–0.29)
	25.0	18.09	0.35	(0.28–0.42)	17.84	0.32	(0.27–0.39)
	27.0	18.80	0.43	(0.33–0.53)	18.59	0.41	(0.32–0.50)
	30.0	19.73	0.54	(0.40–0.67)	19.57	0.52	(0.39–0.65)
	35.0	20.95	0.68	(0.50–0.81)	20.90	0.67	(0.50–0.81)
6.0	18.5	14.06	0.04	(0.03–0.07)	13.85	0.04	(0.02–0.06)
	23.0	16.52	0.23	(0.19–0.28)	16.32	0.21	(0.17–0.25)
	25.0	17.52	0.39	(0.32-0.47)	17.33	0.36	(0.29–0.43)
	27.0	18.45	0.57	(0.46–0.67)	18.28	0.54	(0.43–0.64)
	30.0	19.76	0.78	(0.66–0.87)	19.61	0.76	(0.63–0.86)
	35.0	21.69	0.95	(0.86–0.98)	21.61	0.94	(0.85–0.97)

A sub study of The Tromsø Study: *Fit Futures N* = 532: 279 boys, 253 girls

Predicted values are calculated with mean gender and mean age at measurement in the models

*BMI* body mass index, *CI* confidence interval, *P* probability

Child BMI: BMI according to the International Obesity Taskforce’s age- and sex-specific cut-off values in children 2–18 years [28]

### Mean BMI and BMI SDS in overweight/obese and thin/normal weight children at different ages

**Fig. 2:**
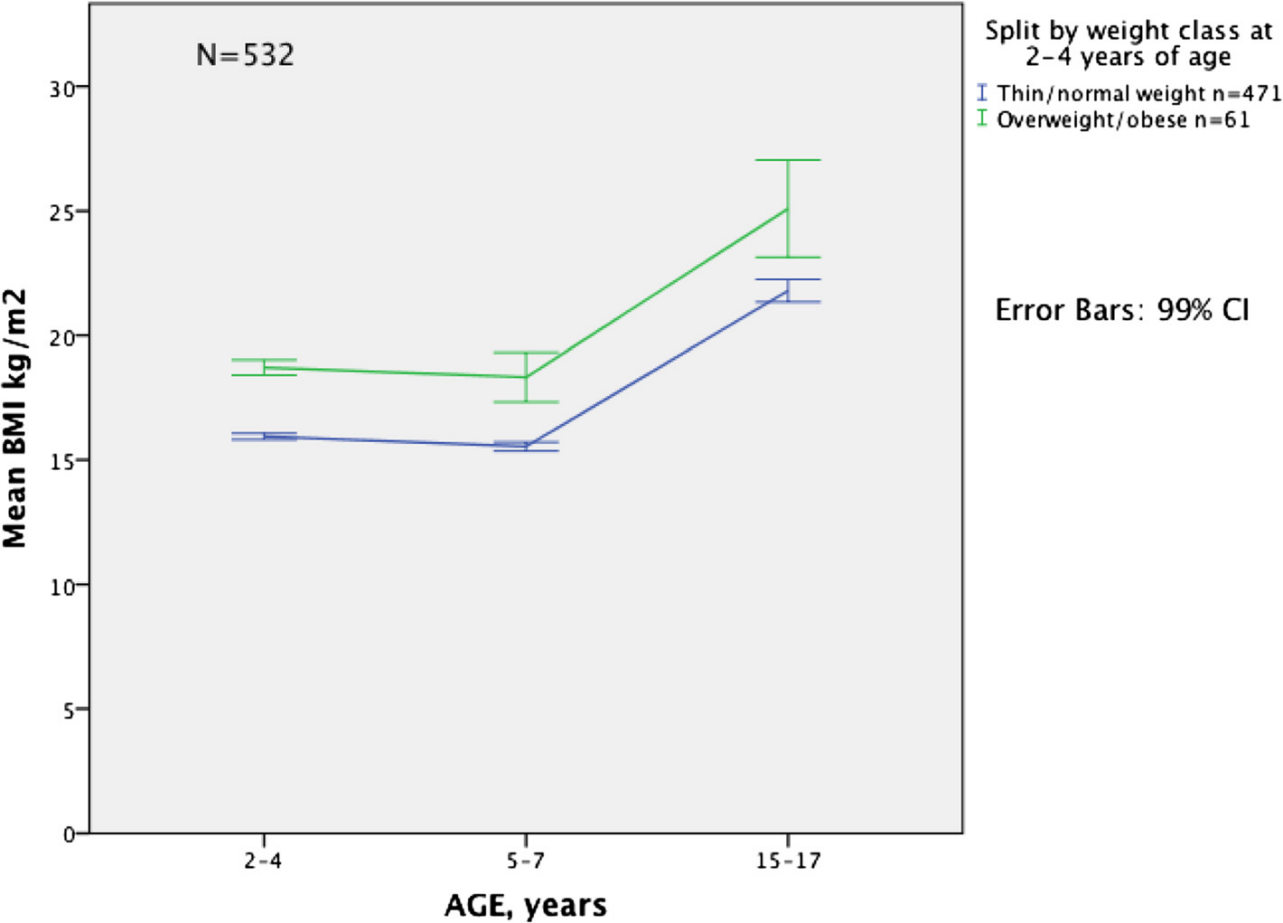
Development in mean BMI from childhood to adolescence according to weight class* at 2–4 years. Mean BMI (kg/m^2^) and 99 % CI at 2–4, 5–7 and 15–17 years of age in thin/normal weight and overweight/obese children at 2–4 years of age. A sub study of The Tromsø Study: *Fit Futures N* = 532. * Weight classes are based on BMI according to the International Obesity Taskforce’s age- and sex-specific cut-off values in children 2–18 years: thinness: adult BMI <18.5 kg/m^2^, normal weight: adult BMI ≥18.5- < 25 kg/m^2^, overweight: adult BMI ≥25- < 30 kg/m^2^, obesity: adult BMI ≥30 kg/m^2^ [28]. BMI: body mass index, CI: confidence interval

**Fig. 3:**
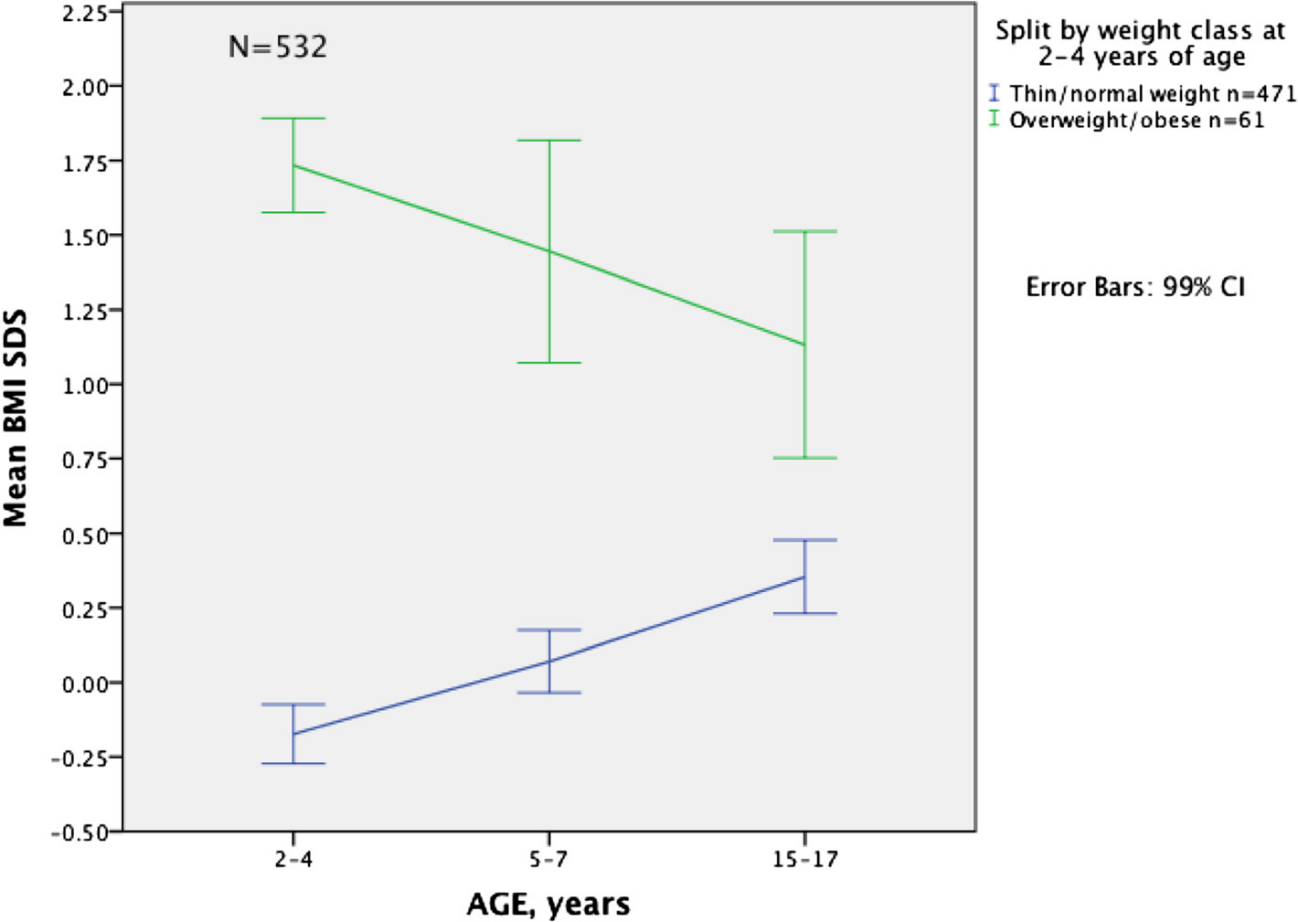
Development in mean BMI SDS from childhood to adolescence according to weight class* at 2–4 years. Mean BMI SDS and 99 % CI at 2–4, 5–7 and 15–17 years of age in thin/normal weight and overweight/obese children at 2–4 years of age. BMI SDS is calculated using LMS values based on an international reference population of children [28]. A sub study of The Tromsø Study: *Fit Futures N* = 532. * Weight classes are based on BMI according to the International Obesity Taskforce’s age- and sex-specific cut-off values in children 2–18 years: thinness: adult BMI <18.5 kg/m^2^, normal weight: adult BMI ≥18.5- < 25 kg/m^2^, overweight: adult BMI ≥25- < 30 kg/m^2^, obesity: adult BMI ≥30 kg/m^2^ [28]. BMI: body mass index, SDS: standard deviation score, CI: confidence interval, IOTF: International Obesity Taskforce, LMS: LMS curves/ LMS method: median (M), coefficient of variation (S) and skewness (L)

Baseline overweight/obese children had a significantly higher mean BMI and BMI SDS also at later ages compared to their thin/normal weight peers (Figs. 2 and 3). The BMI of children who were overweight/obese at 5–7 years of age increased significantly more between 5–7 and 15–17 years than did that of their thin/normal weight peers (8.10 vs. 6.03 kg/m^2^ Mann–Whitney *U* test *p* = 0.001) (Table ***5***). In contrast, among children who were thin/normal weight at 2–4 years of age or at 5–7 years of age, mean BMI SDS increased with age. Among their overweight/obese peers, mean BMI SDS decreased (Table ***5*** and Fig. 3). The same pattern in development of BMI SDS was present when comparing with another reference population, the 1990 UK reference population (Additional file 1: Table S1).

**Table 5:** BMI, BMI standard deviation score (SDS)^a^ and changes during childhood/adolescence according to weight class^b^

Age/ age interval	Thin/normal weight at 2–4 years of age (*n* = 471)		Overweight/obese at 2–4 years of age (*n* = 61)			Thin/normal weight at 5–7 years of age (*n* = 459)		Overweight/obese at 5–7 years of age (*n* = 73)		
	Mean	(SD)	Mean	(SD)	p-value^‡^	Mean	(SD)	Mean	(SD)	p-value^‡^
BMI kg/m^2^										
2–4 years	15.94	(1.06)	18.71	(0.89)	<0.001	−	−	−	−	
5–7 years	15.54	(1.50)	18.32	(2.92)	<0.001	15.27	(1.01)	19.53	(2.29)	<0.001
15–17 years	21.80	(3.82)	25.09	(5.74)	<0.001	21.31	(3.08)	27.63	(5.95)	<0.001
Change in BMI kg/m^2^										
to 5–7 years	−0.40	(1.29)	−0.39	(2.74)	0.051^†^	−	−	−	−	
to 15–17 years	5.85	(3.66)	6.38	(5.73)	0.66	6.03	(2.76)	8.10	(4.84)	0.001
BMI SDS										
2–4 years	−0.17	(0.84)	1.73	(0.47)	<0.001	−	−	−	−	
5–7 years	0.07	(0.88)	1.45	(1.10)	<0.001	−0.06	(0.70)	2.03	(0.72)	<0.001
15–17 years	0.35	(1.04)	1.13	(1.12)	<0.001	0.25	(0.95)	1.69	(1.01)	<0.001
Change in BMI SDS										
to 5–7 years	0.24	(0.78)	−0.29	(1.02)	<0.001	−	−	−	−	
to 15–17 years	0.53	(1.11)	−0.60	(1.14)	<0.001	0.30	(0.85)	−0.34	(0.84)	<0.001

A sub study of The Tromsø Study: *Fit Futures N* = 532: 279 boys, 253 girls

^a^BMI SDS is calculated using LMS values based on an international reference population of children [28]

^b^Weight classes are based on BMI according to the International Obesity Taskforce’s age- and sex-specific cut-off values in children 2–18 years: thinness: adult BMI <18.5 kg/m^2^, normal weight: adult BMI ≥18.5- < 25 kg/m^2^, overweight: adult BMI ≥25- < 30 kg/m^2^, obesity: adult BMI ≥30 kg/m^2^ [28]

^‡^Mann–Whitney *U* test for comparing groups. Monte Carlo sig. (2.tailed) confidence level 99 %

^†^CI for mean change in the two groups: −0.40 (CI: −0.58 - -0.23) -0.39 (CI: −1.46 - 0.68)

*BMI* body mass index, *LMS* LMS curves/ LMS method: median (M), coefficient of variation (S) and skewness (L), *SD* standard deviation, *SDS* standard deviation score

## Discussion

In this population-based study using longitudinal data, the prevalence of overweight/obesity was 11.5–13.7 % in childhood, and increased to 20.1 % in adolescence. These rates in childhood are comparable with rates reported in other studies from Norway and Nordic countries [6, 12, 22, 35], but lower than rates from Norwegian children born after the year 2000 [4, 23], rates reported from Southern European countries [2], and the United States [36]. For adolescents, the rates were almost twice as high as rates from Western-Norway [35], but approximately the same as those in Mid-Norway and other Nordic countries [12, 22, 37] and lower than rates from Southern European countries [2]. The difference in prevalence rates in Norway may be due to differences in prevalence between birth cohorts. Children in our cohort were born in 1992–1994. Cohorts with children born after 2000 have reported higher prevalence rates [23]. The possibility of selection bias in our study, or the study from Western-Norway [35, 38] cannot be ruled out, also as discussed in their paper. Participation proportion in the comparable study was 45 % among adolescents in the upper-secondary school group. The present study included 55 % of adolescents under 18 years in *Fit Futures 1*. Other suggested explanations have been related to differences between urban and rural areas with higher prevalence in more rural areas [39]. Tromsø is the largest city in North-Norway, however the municipality consists of both urban and more rural areas. Differences in socio-economic status, such as parental educational level and income are other explanations [5, 35, 39]. Unfortunately we lack individual information on socio-demographic factors.

The majority of children remained thin/normal weight between childhood and adolescence. However, we found a moderate indication of tracking of overweight/obesity from childhood to adolescence, as well as from 2–4 years of age to 5–7 years of age. Results from the different tracking analyses were consistent. The strength of the association was strongest between 2–4 and 5–7 years of age (mean time interval 3.4 years). Tracking coefficients were of similar magnitude between 5 and 7 years of age and adolescence (mean time interval 10.6 years) and may be considered a stronger indication of tracking because the risk factor was stable over a longer time interval. Glavin et al. found very high OR for being overweight or obese at 8 years of age among children who were overweight or obese at 4 or 6 years of age (63.8, 95 % CI: 45.5–91.5 and >100, 95 % CI: 90.9- > 100, respectively) [23]. Tracking coefficients are influenced by the time interval considered, and a higher tracking coefficient over a short period isn’t necessarily a stronger indication of tracking than a lower coefficient over a longer time interval [11, 16]. Therefore we consider the findings of moderate tracking coefficients from 2–4 and 5–7 years of age to adolescence to be consistent and important findings in our study. The same pattern has also been found in longitudinal studies from Sweden and Iceland [12, 22]. Our results are similar to the results of the Swedish study, in which 60 % of children who were overweight or obese at 5.5 years, and 44 % of children who were overweight or obese at 2.5 years were also overweight or obese at 20 years of age [22].

Although we found a moderate degree of tracking from 2–4 and 5–7 years of age to 15–17 years age, the proportion of overweight/obese children that became thin/normal weight was higher (60.7 % and 37.0 %) than the proportion of thin/normal weight children that became overweight/obese (17.6 % and 13.3 %) in the same time interval. This finding is in accordance with some studies [12, 22], but not others [20, 23]. Recent systematic reviews and a meta-analysis [10, 11] concluded that reliable studies consistently reported an increased risk for overweight and obese youths to become overweight or obese adults. They further reported that there was strong evidence that persistence of overweight and obesity is moderate. The findings in our study are in line with this conclusion, although the use of different definitions of overweight and obesity makes direct comparisons challenging. BMI at 2–4 years of age was a poor predictor of overweight/obesity at 15–17 years of age. At 2.5 years of age only BMI corresponding to an adult BMI of 30 kg/m^2^ or higher, predicted overweight/obesity at adolescence with over 50 %. High BMI at 5–7 years of age was a better predictor of overweight/obesity in adolescence with higher probabilities. More severe overweight and obesity in childhood seems to be a stronger predictor of overweight/obesity later in life. This is in accordance with findings in several other studies [10, 11, 22, 36].

Mean BMI in our study decreased between 2–4 and 5–7 years of age, but increased between both ages in childhood and adolescence. This may be explained by the natural variation in BMI during early childhood and the adiposity rebound that occurs between 3 and 7 years of age that is expected in the natural development of BMI [19, 28]. Mean BMI increase in children who were overweight/obese at 5–7 years of age, was significantly higher than that of thin/normal weight children. Change in mean BMI in children who were overweight/obese at 2–4 years of age did not differ significantly compared to thin/normal weight children. This shows the same pattern as the tracking analyses, i.e. that overweight and obesity tend to be more stable later in childhood and overweight and obesity may get more severe with age [11, 22, 36]. In our study the number of obese participants was too low to do separate analyses for the obese group.

Our results regarding the development of BMI differ from those on the development of BMI SDS, as BMI SDS decreased in overweight/obese children and increased in the thin/normal weight children in the same time interval. This indicates that both groups transitioned to more normal weight over time compared to the IOTF reference population [28]. This may also be seen as an expression of the statistical phenomenon; the regression towards the mean that may occur in repeated measurements [40]. Other studies that have looked at the development of overweight and obesity retrospectively have found opposite results [20, 23]. BMI SDS is commonly used to look for long-term trends in growth [6, 23, 28] and we wanted to look at the natural development prospectively. This may be a basis for comparison for intervention studies. It has been suggested that short-term change in adiposity in children is best evaluated by BMI and that BMI SDS is less suitable because it depends on baseline BMI [25, 26]. The children in our study were not severely overweight/obese at baseline. The prevalence of obesity was 1.5 % (*n* = 8) and 4.3 % (*n* = 23) in our study sample (boys and girls combined), with a BMI SDS of 1.73 and 2.03 among the overweight/obese at 2–4 and 5–7 years of age, respectively. The weight classes thinness and normal weight were merged in our study, which may explain the increase in BMI SDS in this combined group as the prevalence of thinness decreased with age. BMI SDS may also depend on the growth reference used. We used the international reference as recommended [24, 28], but we also repeated the analyses using the 1990 UK reference population [30]. No difference in development of BMI SDS was present when comparing our data with the 1990 UK reference population [30]. The pattern shown in Fig. 3 might be differences in growth pattern between different populations since Norway, and in general Nordic populations, have lower prevalence of overweight and obesity than other populations.

More girls than boys were categorized as overweight/obese in childhood. At adolescence this tendency changed and more boys than girls were categorized as overweight/obese. This is in accordance with findings from several other studies [4–6, 22, 35] and is an interesting finding that should be explored in further research. However, the gender difference was statistically significant only at 5–7 years of age and was not investigated further in this study.

Strengths of this study were the population-based design, the high participation proportion in *Fit Futures 1* (>92 %) [27], longitudinal data over more than a decade, and a fairly large study sample for the tracking analyses (*n* = 532). The prevalence rate of overweight/obesity was comparable with most other study populations in Norway and the Nordic countries born before 2000. Excluded youths, in the core age group under 18 years of age, had statistically significantly higher BMI at adolescence (boys: 22.7 kg/m^2^, girls: 22.6 kg/m^2^) compared to the study sample (boys: 22.1 kg/m^2^, girls: 22.2 kg/m^2^). Nevertheless we consider the groups to be essentially the same, as the differences were small [27]. We therefore consider the results to be representative for Norwegian children and adolescents. Data collected in *Fit Futures 1* were standardized measures and a calibrated scale was used, therefore the data are considered valid [27]. In this study we used IOTF cut-off values and LMS values based on an international reference population, as recommended [28]. This may ease comparisons between studies and is an advantage of our study. We also believe that the use of several methods showing consistency between findings strengthens the reliability of our findings.

However, our study also has some limitations. The magnitude of the tracking coefficient can be affected by inaccuracies in the measures of height and weight collected from childhood health records. Several factors can affect this accuracy e.g. inter-observer variability and the accuracy and quality control of different scales [41]. Usually these factors are anticipated to be non-differential errors that lead to both higher and lower height and weight measures, and that thus affect all weight classes in approximately equal amounts [5, 6]. Data from routine measurements are commonly used in studies of overweight and obesity in children [5, 6, 12, 22, 23]. Despite the fairly large study sample, the obese group was too small to investigate any differences between the obese group and the other weight classes separately. Other studies have indicated that the degree of tracking is higher with more severe overweight and obesity [11, 22]. Another weakness of this study is the lack of explanatory variables that were available from childhood. This gives us limited possibilities to explain and adjust our findings for influential factors that have been identified in other studies, like maternal BMI, maternal smoking, parental education level and other factors [35, 42]. This is especially a limitation with regard to our description of the natural development of BMI. We don’t have information of potential influential factors like dietary habits, physical activity level or any weight controlling interventions in this period from childhood to adolescence.

This study adds to the knowledge base of tracking of overweight and obesity using data from early childhood to adolescence in a Norwegian cohort. From a public health perspective, the positive finding was that the vast majority of normal weight children remained of normal weight up to adolescence. Since high BMI alone, especially at 2–4 years of age, only is of moderate predictive value for overweight and obesity at adolescence, additional risk factors as parental factors must be taken into account to identify children at high risk [35, 42]. Children at high risk of becoming overweight/obese in later life may be identified before they reach school age. Early childhood and preschool age stands out as an important time to focus both on individual preventive initiatives targeting those at high risk as well as all children. The need for early intervention has also been pointed out by other researchers [43]. Childhood health controls are a natural meeting point between public health nurses, children, and their parents. Our study also showed that the prevalence of overweight and obesity increased significantly (doubled in boys) between childhood and adolescence. In addition, children changed weight classes in both directions. Therefore a broad focus on general preventive efforts in society, targeting all children, seems highly appropriate.

## Conclusion

The prevalence of overweight and obesity increased with age. We found a moderate indication of tracking of overweight/obesity from childhood to adolescence, with stronger associations between 5–7 and 15–17 years of age than between 2–4 and 15–17 years of age. Six out of 10 children who were overweight/obese at 5–7 years of age were overweight/obese at 15–17 years of age. Preventive initiatives addressing overweight/obese children at high risk should start in early childhood, before 5–7 years of age. However, since high childhood BMI alone only is a moderate predictor of overweight or obesity later in life, general preventive efforts targeting all children are most important.

## Additional file

Additional file 1: Table S1. BMI standard deviation score (SDS)* and changes during childhood/adolescence using a UK reference population. (DOC 37 kb)

## Abbreviations

BMI: body mass index
CI: confidence interval
IOTF: International Obesity Taskforce
LMS: LMS curves/ LMS method: median (M), coefficient of variation (S) and skewness (L)
OR: odds ratio
SDS: standard deviation score
UK: United Kingdom
