# Supplementary material for: Tracking of overweight and obesity from early childhood to adolescence in a population-based cohort – the Tromsø Study, Fit Futures
Source: BMC Pediatr. 2016 May 10;16:64. doi: 10.1186/s12887-016-0599-5 (PMC4863357; doi:10.1186/s12887-016-0599-5)
Supplement: Additional file 1: Table S1. — BMI standard deviation score (SDS)* and changes during childhood/adolescence using a UK reference population. (DOC 37 kb) [file 12887_2016_599_MOESM1_ESM.doc]

## Additional file 1: Table S1 - BMI standard deviation score (SDS)* and changes during childhood/adolescence using a UK reference population

| Age/ age interval |  | Thin/normal weight** at 2-4 years of age (n=471) | | Overweight/obese** at 2-4 years of age (n=61) | |  |  | Thin/normal weight** at 5-7 years of age (n=459) | | | Overweight/obese** at 5-7 years of age (n=73) | | | |  | |
| --- | --- | --- | --- | --- | --- | --- | --- | --- | --- | --- | --- | --- | --- | --- | --- | --- |
|  |  | Mean | (± SD) | Mean | (± SD) | p-value **^‡^** |  | Mean | | (± SD) | Mean | | (± SD) | | | p-value **^‡^** |
| BMI SDS |  |  |  |  |  |  |  |  | |  |  | |  | | |  |
| 2-4 |  | −0.26 | (0.86) | 1.68 | (0.46) | <0.00 1 |  | − | | − | − | | − | | |  |
| 5-7 |  | −0.09 | (0.93) | 1.35 | (1.18) | <0.001 |  | −0.23 | | (0.75) | 1.96 | | (0.79) | | | <0.001 |
| 15-17 |  | 0.28 | (1.13) | 1.12 | (1.22) | <0.001 |  | 0.16 | | (1.02) | 1.73 | | (1.11) | | | <0.001 |
| Change in BMI SDS | |  |  |  |  |  |  |  | |  |  | |  | | |  |
| to 5-7 |  | 0.17 | (0.82) | −0.34 | (1.08) | <0.001 |  | − | | − | − | | − | | |  |
| to 15 -17 |  | 0.54 | (1.18) | −0.56 | (1.23) | <0.001 |  | 0.39 | (0.91) | | | −0.24 | | (0.92) | <0.001 | |

A sub study of The Tromsø Study: *Fit Futures* N=532: 279 boys, 253 girls.

Change in BMI SDS^*^ between time points in children categorized as thin/normal weight^**^ or overweight/obese^**^ at 2-4 years of age.

***** BMI SDS is calculated using LMS values from a UK reference population of children [30]

** Weight classes are based on BMI according to the International Obesity Taskforce’s age- and sex-specific cut-off values in children 2-18 years: thinness: adult BMI <18.5 kg/m^2^, normal weight: adult BMI ≥18.5-<25kg/m^2^, overweight: adult BMI ≥25-<30 kg/m^2^, obesity: adult BMI ≥30 kg/m^2^ [28].

**^‡^** Mann-Whitney U test for comparing groups. Monte Carlo sig. (2.tailed) confidence level 99%

BMI: body mass index, LMS: LMS curves/ LMS method: median (M), coefficient of variation (S) and skewness (L), SD: standard deviation, SDS: standard deviation score, UK: United Kingdom.
